# Exploring hepatic hormone actions using a compilation of gene expression profiles

**DOI:** 10.1186/1472-6793-5-8

**Published:** 2005-06-13

**Authors:** Nina Ståhlberg, Roxana Merino, Luis Henríquez Hernández, Leandro Fernández-Pérez, Albin Sandelin, Pär Engström, Petra Tollet-Egnell, Boris Lenhard, Amilcar Flores-Morales

**Affiliations:** 1 Department of Molecular Medicine, Karolinska Institute, 17176 Stockholm, Sweden; 2 Health Sciences Center, Pharmacology Section, Las Palmas de GC University – Instituto Canario de Investigación del Cancer – RTICCC, 35080 – Las Palmas de GC, Spain; 3 Center for Genomics and Bioinformatics, Karolinska Institute, 17176 Stockholm, Sweden

## Abstract

**Background:**

Microarray analysis is attractive within the field of endocrine research because regulation of gene expression is a key mechanism whereby hormones exert their actions. Knowledge discovery and testing of hypothesis based on information-rich expression profiles promise to accelerate discovery of physiologically relevant hormonal mechanisms of action. However, most studies so-far concentrate on the analysis of actions of single hormones and few examples exist that attempt to use compilation of different hormone-regulated expression profiles to gain insight into how hormone act to regulate tissue physiology. This report illustrates how a meta-analysis of multiple transcript profiles obtained from a single tissue, the liver, can be used to evaluate relevant hypothesis and discover novel mechanisms of hormonal action. We have evaluated the differential effects of Growth Hormone (GH) and estrogen in the regulation of hepatic gender differentiated gene expression as well as the involvement of sterol regulatory element-binding proteins (SREBPs) in the hepatic actions of GH and thyroid hormone.

**Results:**

Little similarity exists between liver transcript profiles regulated by 17-α-ethinylestradiol and those induced by the continuos infusion of bGH. On the other hand, strong correlations were found between both profiles and the female enriched transcript profile. Therefore, estrogens have feminizing effects in male rat liver which are different from those induced by GH. The similarity between bGH and T3 were limited to a small group of genes, most of which are involved in lipogenesis. An *in silico *promoter analysis of genes rapidly regulated by thyroid hormone predicted the activation of SREBPs by short-term treatment *in vivo*. It was further demonstrated that proteolytic processing of SREBP1 in the endoplasmic reticulum might contribute to the rapid actions of T3 on these genes.

**Conclusion:**

This report illustrates how a meta-analysis of multiple transcript profiles can be used to link knowledge concerning endocrine physiology to hormonally induced changes in gene expression. We conclude that both GH and estrogen are important determinants of gender-related differences in hepatic gene expression. Rapid hepatic thyroid hormone effects affect genes involved in lipogenesis possibly through the induction of SREBP1 proteolytic processing.

## Background

The completion of human and rodent genome sequences [1-3] has brought the post-genomic era to the field of endocrine research. Detailed genetic maps of the main endocrine models can now be used to study the molecular basis of endocrine disease and the molecular mechanisms of hormone actions. The possibility to explore expression data of thousands of genes across multiple experimental paradigms promise to rapidly increase our understanding of biological systems [4,5]. The acquisition of experimental data at a genomic scale requires high throughput technologies such as DNA microarray analysis. Microarrays enable the simultaneous assessment of expression levels of tens of thousands of gene products in an ease to perform assay. Microarrays are especially attractive to the field of endocrine research because regulation of gene expression is an important mechanism whereby hormones exert their physiological actions. This is obvious in the case of steroid and thyroid hormones, which use intracellular receptors belonging to the nuclear receptor family of transcription factors [6]. Peptide hormones also regulate gene expression after activating complex cascades of intracellular signaling events upon binding to transmembrane receptors [7]. If the relation between hormones and the expression of different genes could be annotated, the abundant knowledge concerning endocrine physiology might be used to clarify the biological function of those genes. On the other hand, because expression profiles are rich in information, they are suitable to study the complex and pleitropic actions of hormones.

Here we analyzed a compilation of rat liver expression profiles from experiments designed to study gender and hormone actions in order to provide novel insight into the mechanisms of action of specific hormones. The dataset used in this study comprises the actions of thyroid hormone (T3), 17-α-ethinylestradiol and GH in liver. The data is freely available from the Endocrinology Gene Expression Database –  and have also been deposited in Gene Expression Omnibus . Using this collection of microarray data, we analyzed the differential contribution of estrogens and GH to the regulation of gender differentiated liver gene expression. We also compared the actions of GH and T3 in liver and found a small overlap comprising genes involved in lipogenesis suggesting the common regulation of SREBP transcription factors. The regulation of SREBP1 by GH and thyroid hormone was analyzed.

## Results

### Exploring hormonal regulation of gene expression in liver

Several expression profiles where compiled and used to obtain insight into the hormonal regulation of liver physiology. The following six experiments were analyzed together (Table 1): 17-α-ethinylestradiol treatment of male rats, infusion of bGH in young (3 month) male rats [8], infusion of hGH in old (2 years) male rats [9], bGH treatment of primary hepatocytes isolated from young male rats, comparison of female and male rats [8], and the rapid effects of T3 treatment of hypothyroid mice [10]. The following questions were formulated: Can expression profiling be used to clarify the physiological actions of hormones in the liver? Can promoter analysis of hormonally regulated genes provide novel insight into the mechanism of hormone actions? It should be noted that our intentions were not to exhaustively explore the data set but rather to illustrate the utility of microarray data mining in endocrine research. The experiments included in this analysis were not specifically designed to answer the questions formulated in the present study although they are sufficient to test our hypotheses. This mimics the situation when the experimental biologist try to derive knowledge from a set of disparate experiments performed in different laboratories, using different experimental designs and microarray technologies. Importantly, we have taken all possible measures to minimize systematic experimental errors. All the arrays used for analysis have been fabricated in house from a unique set of PCR products and these have been validated in numerous studies [7-15]. The protocols for labeling and data analysis were also similar along all the experiments. Within each of the experiments included in this analysis, we have accounted for biological variability by independent replication of the measurements using RNA from individual animals.

**Figure 1:**
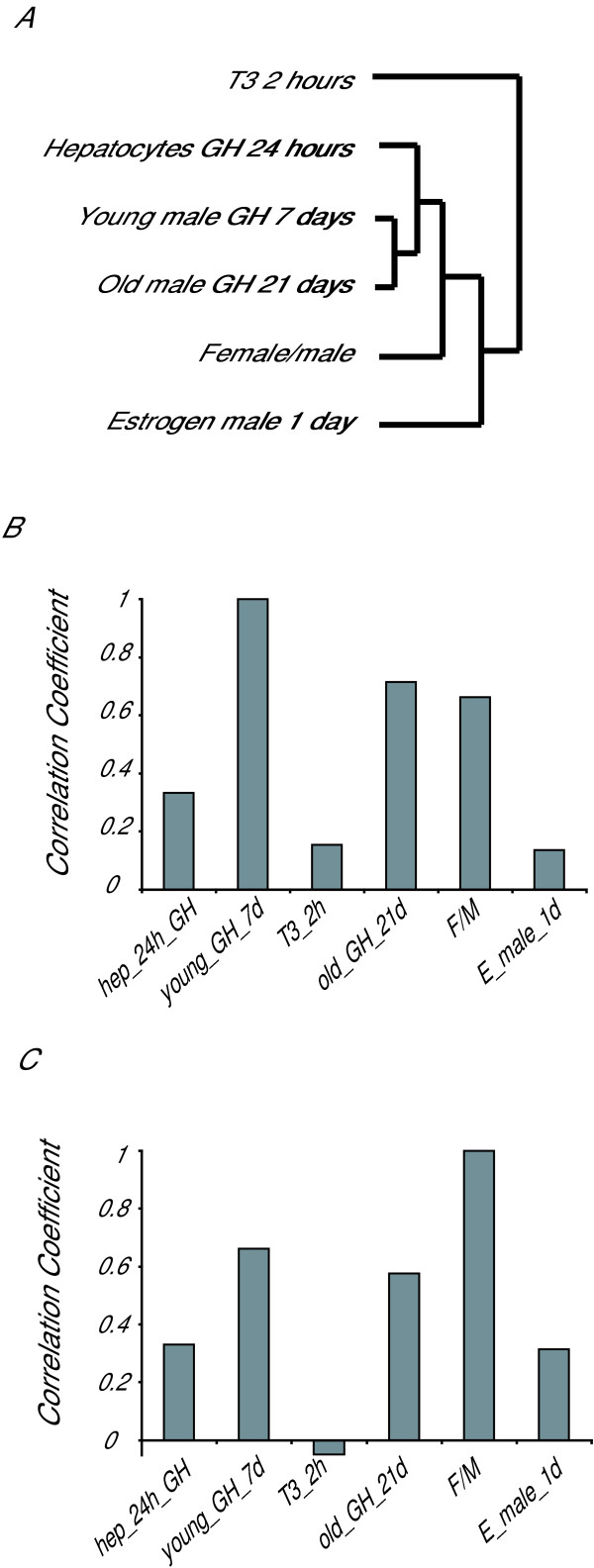
**A comparison of all experiments included in the study**. a) Hierarchical clustering of hepatic gene expression profiles using average linkage analysis and the Euclidean distance metric clusters together the *in vivo *experiments concerning GH. The included experiments were: E_male_5d: 17-α-ethinylestradiol treatment of male rats for 1 day, young_GH_7d: infusion of bovine growth hormone (bGH) in young (3 months) male rats for 7 days, old_GH_21d: infusion of human growth hormone (hGH) in old (2 years) male rats for 3 weeks, hep_GH_24h: bGH treatment of primary hepatocytes from young (2 months) male rats for 24 hours, F/M_rat: comparison of untreated female and malerat livers, and T3_2h: thyroid hormone treatment of hypothyroid mice for 2 hours. b) Correlation coefficients of the different expression profiles to the profile of young GH-treated male rats. The strongest correlation is found between GH-treated young and old male rats, and also between young males treated with GH and female rats. The *in vitro *GH-treatment of isolated primary rat hepatocytes is also similar to the *in vivo *situation. c) Correlation coefficients of the different expression profiles to the female/male comparison. The strongest correlation is found between the female profile and GH-treated young and old male rats. Estrogen treatment of male rats shows lower correlation to gender.

Unsupervised clustering algorithms were first used on the entire dataset to gain a global view of different hormone actions in liver. Average-linkage hierarchical clustering (using Euclidean distance as measurement of similarity) was used to evaluate the relation between the expression profiles. As shown in Figure 1a, the effects induced by GH treatment in young males were similar to those induced by GH treatment in old animals. This observation supports the robustness of the GH effects since the designs of the two experiments differ not only regarding the age of the animals but also in the dose and type of hormone administered (bovine versus human GH). A calculation of the correlation coefficients between bGH-induced expression changes and the rest of the experiment groups supports this conclusion (Figure 1b). Interestingly, the expression changes induced by GH treatment of primary rat hepatocytes cultured on matrigel show a positive, although small, correlation with the effects observed *in vivo *(Figure 1a and 1b). This suggests that hepatocytes significantly contribute to the expression changes measured in intact liver upon GH treatment. The fact that well-known GH-regulated genes: insulin-like growth factor 1(IGF-1) and CYP2C12 [16] are also regulated in hepatocytes helps to substantiate our conclusion. The similar effects on gene expression after *in vivo *or *in vitro *GH treatment were confirmed for some genes using quantitative real-time PCR (Tables 2 and 3).

As shown in figure 1b, the correlation between GH treatment and thyroid hormone or estrogen treatment in young males was rather low. This is not surprising since both hormones have distinct liver functions not always overlapping those of GH. Differences in expression can also arise from the choice of treatment duration, dose and mode of hormone treatment and this could result in the underestimation of commonly regulated genes. This ambiguity can only be resolved by measuring more expression profiles in experiments specifically designed to study hormonal interactions.

### GH and estrogen contribute to gender differences in hepatic gene expression

When we compared the gender-related expression differences to the rest of the experiments (Figure 1c, Table 4), it was evident that as many as 48% of the female-enriched transcripts were also up-regulated by continuous GH infusion in 3 month old males. Similarly, around 34% of the male-enriched transcripts were down-regulated in males by the same treatment. Few genes (less than 4% of the male-enriched and none of the female-enriched) were affected in the opposite direction by continuous treatment with GH. On the other hand, estrogen treatment in 3 month old male rats induced the expression of 27% of the female-enriched genes, and repressed the expression of 27% of the male-enriched ones (Table 5). Again, few genes (less than 7% of the gender differentiated) were affected in the opposite direction by treatment with estrogen. This data strongly suggest that both estrogen and GH significantly contribute to the gender differences in adult rat liver. Although, as illustrated in Figure 1c, one week of continuous infusion with GH is more efficient than an injection with 17-alpha-ethynilestradiol to feminize the adult male rat liver expression profile.

### T3 and GH regulate lipogenic genes in liver

Both GH and T3 are required for longitudinal growth. Therefore, we expected these hormones to have some overlapping effects on liver gene expression. Nevertheless, we found that similarities were limited to a small number of genes (indicated with a # in Table 6). A comparison of frequency distribution among gene ontology (GO) categories related to the biological function between the whole set of expressed genes and those up-regulated by T3 and GH revealed a statistically significant overrepresentation (p < 0.05) of regulated genes in the category of lipid metabolism. Similar overrepresentation of genes involved in lipogenesis was identified among the genes induced by thyroid hormone.

**Figure 2:**
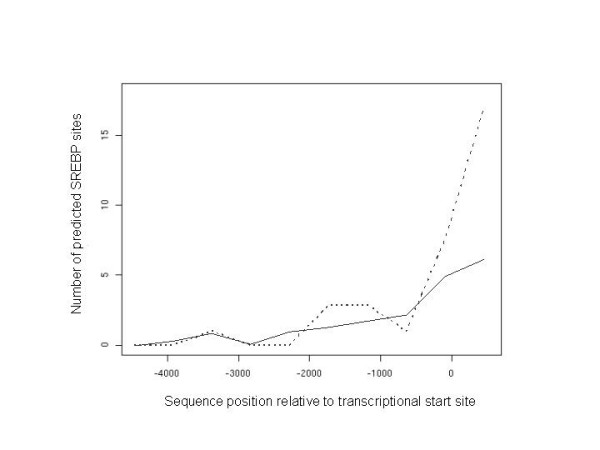
**Promoter analysis of T3-regulated genes**. The promoter analysis revealed an overrepresentation of putative SREBP binding sites in the T3-regulated group compared to the control group. The figure shows the expected number of SREBP binding sites in each region of the promoter as estimated from the control group (solid line), and the observed number in the T3-regulated group (dotted line). A Chi-square test showed very significant (p = 0.0001) overrepresentation of SREBP sites in the region surrounding the transcriptional start site. The genes included in this analysis are indicated with a * in Table 6.

The SREBP family of transcriptional regulators plays an essential role in the regulation of lipogenesis and is known to regulate several of the genes found to be induced by thyroid hormone (Table 6) [17]. Because the transcriptional effects of T3 were rapid, we hypothesize the existence of a direct mechanistic crosstalk between SREBPs and T3 signaling in mouse liver. Therefore, we analyzed the promoter regions of T3-induced genes to find out whether any consensus SREBP binding sites could be found in promoter regions. The genes that were included in this analysis are indicated with a * in Table 5. Interestingly, we could identify a clear overrepresentation of putative SREBP binding sites around the transcriptional start site in the T3-regulated group compared to the control group (Figure 2). To make sure that this result was not only due to higher phylogenetic conservation in the T3-regulated group, a basic statistic analysis was performed: the region from -500 to +100 relative the transcriptional start sites (TSS) was picked out of each alignment in both groups. A simple two-sided t-test of the mean conservation in both groups showed that they were not significantly different (p = 0.12). In fact, the T3-regulated group had a slightly lower (but not statistically significant) mean degree of conservation than the control group. We tested if the distribution of SREBP sites was significantly different between the two groups using Chi-square analysis. As the number of genes in the control group was higher, this distribution was normalized to correspond to 30 genes instead of the original 300 controls. The test demonstrates conclusively (p = 0.0001) that the two distributions are different and thus that there is a clear overrepresentation of putative SREBP binding sites near the TSSs in the T3-regulated group (Figure 2). In contrast, we were not able to localize a significant overrepresentation of thyroid hormone response elements (TREs) in the close promoters of the T3-regulated genes. We can not exclude that TREs exist in the promoters of these genes, but either they are located further away from the transcriptional initiation sites or in areas that are not phylogenetically conserved between mice and humans.

**Figure 3:**
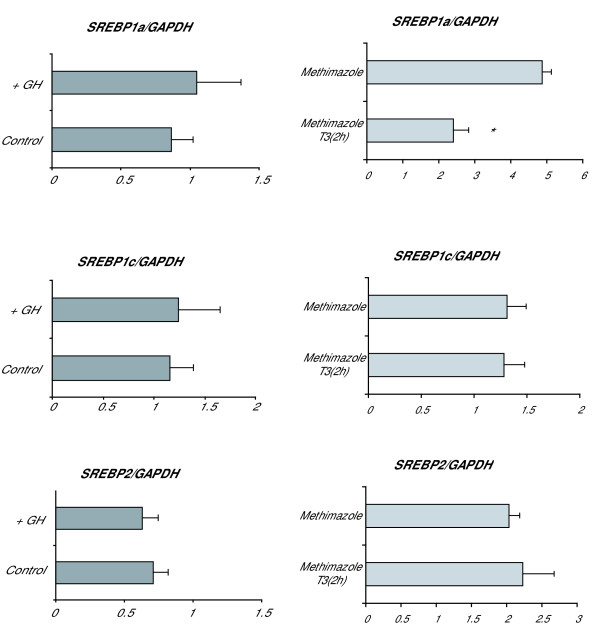
**Effects of GH and T3 on SREBP expression measured by real-time PCR**. Two hours after thyroid hormone treatment of hypothyroid mice, SREBP1a expression is down-regulated, while SREBP1c and 2 are unaffected. Expression of SREBP1a, 1c and 2 is not affected after 7 days of bGH infusion in young male rats. * p < 0.005.

Three different SREBP isoforms, 1a, 1c and 2, encoded by two distinct genes have been described. SREBP1c is the predominant form in adult liver and adipocytes [17]. SREBPs are translated as large precursors tethered to the endoplasmic reticulum and nuclear membrane where they, in response to sterol depletion, are proteolytically cleaved into mature, transcriptionally active factors that migrate to the nucleus and bind sterol regulatory elements of specific genes [18]. The mechanisms whereby T3 regulate SREBP actions have been previously studied but remain unclear. Long-term treatment with T3 has been shown to induce SREBP2 expression in hepatocytes [19]. We tested whether rapid transcriptional induction could account for the observed up-regulation of lipogenic genes. As shown in Figure 3, T3 treatment had no effect on SREBP1c or SREBP2 mRNA levels whereas the expression of SREBP1a was significantly reduced. The results demonstrate that rapid transcriptional induction of SREBPs is not the mechanism behind the T3 regulation of lipogenic genes. Since the set of genes overlapping between T3 and GH contained mostly genes involved in lipid metabolism, we also analyzed the transcriptional induction of SREBP1a, 1c and 2 after long term GH treatment, but failed to detect any significant effect that could explain the up-regulation of lipogenic genes (Figure 3).

**Figure 4:**
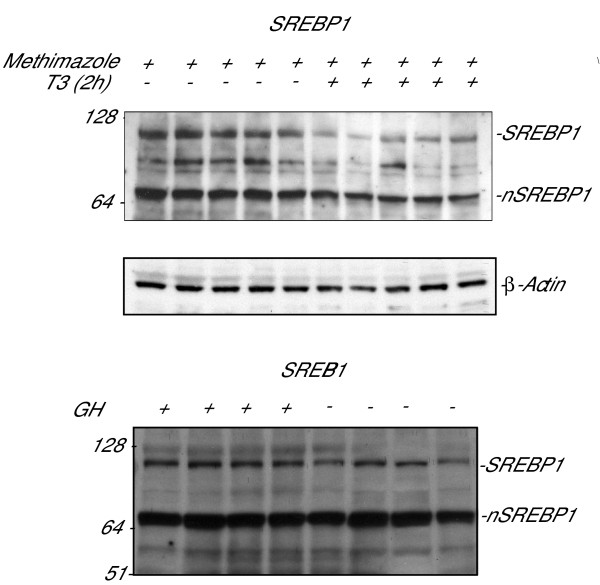
**Effects of GH and T3 on SREBP1 processing**. A Western blot showing hypothyroid mice livers with or without a T3-injection 2 hours prior to measurement. In the T3-treated samples, there is a decreased concentration of high molecular weight (ER bound) SREBP1, indicating an activation of its proteolytic processing by T3.

We next explored whether early events in SREBP activation, such as the proteolytic processing of endoplasmic reticulum (ER) resident SREBPs could be regulated by T3. The ER and nuclear forms of SREBP1 were measured before and after T3 treatment. The analysis was extended to study the effects of GH treatment. The Western blots in Figure 4 show a decrease in the concentration of ER bound (high molecular weight) SREBP1, indicating a rapid activation of its proteolytic processing by T3. No significant effect of T3 could be detected for SREBP2 (data not shown). Interestingly, the concentration of the nuclear (short) form of SREBP1 was also reduced indicating that the total nuclear concentration of SREBP1 poorly reflects its activity as judged by the transcriptional induction of target genes. In contrast to the findings with thyroid hormone, the effect of GH doesn't seem to be exerted through similar mechanisms since no significant changes in ER or nuclear SREBP levels were evident after GH treatment (Figure 4). It is therefore possible that alternative mechanisms such as RNA stability or even the activation of other transcription factors account for the GH effects on lipogenic genes.

## Discussion

Here, we have attempted an analysis of a compilation of expression profiles to gain insight into the hormonal regulation of liver gene expression. We demonstrated that a positive correlation exists between the effects of GH treatment in primary hepatocytes cultured on matrigel and those detected *in vivo*. Nevertheless, the correlation was not very high despite the care taken of cultivating the hepatocytes on matrigel to avoid de-differentiation. We know from previous studies that hepatocytes cultured on matrigel express GH receptors, that GH signalling through the JAK2/STAT5 pathway is functional and that GH induces IGF-1, a well- known GH regulated gene *in vivo *[25]. Therefore, the differences between the *in vivo *and *in vitro *models are likely due to structural and systemic factors found in intact liver which would be required for the full extension of GH actions. On the other hand, our data demonstrate that primary hepatocytes cultured on matrigel do provide a model to study some GH activated mechanisms; those directly related to the activation of the GH receptor and its signaling molecules. The newly described GH regulated genes in hepatocytes (Table 3): phytanoyl-CoA hydroxylase (Phyh), hydroxysteroid 11-beta dehydrogenase 1 (Hsd11b1), the catalytic subunit of protein phosphatase 3, alpha isoform (Ppp3ca) and fatty acid translocase/CD36 antigen (FAT/CD36) constitute new target genes that could be used to study the basis of transcriptional regulation by GH in hepatocytes.

Our global assessment of gene expression, demonstrates that estrogen and to a larger extent, the female-like continuous pattern of GH secretion are important for the maintenance of the gender differences in liver gene expression (Tables 4 and 5). These data support the existence of a cross talk in the hepatic actions of GH and estrogens for the regulation of a subset of the female-enriched genes. The exact mechanism of this cross talk cannot be extracted from the data, but previous findings offer a possible explanation. It is well known that the liver expresses relatively low levels of estrogen receptor and that some estrogen-induced effects in liver, including the expression of the estrogen receptor itself, may be secondary to its feminizing effect on GH pituitary secretion [20,21]. The role of GH secretory patterns in determining gender differences in rat liver expression of genes involved in sterol and drug metabolism has been described before [16,22]. Here and in recent studies by Ahluwalia et al [23] and Stahlberg et al [8], the significance of this regulation is demonstrated at a more comprehensive level and novel hepatic gender-regulated genes are identified. We don't know yet how gender determines the transcription of these genes. To the date, only a few gender-predominant and GH-regulated transcription factors have been identified, including female predominant HNF-6 [24] and male predominant STAT5b [25]. Nevertheless, an analysis of promoter sequences of the genes identified in Table 4 has failed to identify any significant overrepresentation of HNF-6 or STAT5 consensus binding sites (data not shown). Therefore, further analyses are required to better understand the molecular mechanism behind the feminizing actions of GH in liver.

Both GH and thyroid hormone are required for longitudinal growth [26]. The coordinate actions of these hormones are achieved through multiple mechanisms. Thyroid hormone is a key activator of GH secretion in the pituitary gland while GH promotes the formation of T3 from less active T4 in peripheral tissues [27]. Through the comparisons of expression profiles, we could identify a small group of genes that were up-regulated by both hormones. Most of the genes were assigned to the category of lipid metabolism by unbiased classification based on the current gene ontology. This is in agreement to the known lipogenic effects of both hormones in liver [7,10,28]. The correlation between GH and T3 effects was small which could be due to important differences in experimental design as well as to mechanistic differences. Therefore, the study of the long term effects of T3 treatment has the potential to identify more overlapping effects.

The effects of T3 described herein are produced after 2 hours of treatment and are likely to be direct hormonal effects on the liver. Therefore, we analyzed the proximal promoter of genes rapidly regulated by T3 to gain further insight into the mechanisms of hepatic T3 actions. Interestingly, we were not able to localize a significant overrepresentation of TREs in the close promoters of the T3-regulated genes. On the other hand, we found a clear overrepresentation of putative SREBP binding sites; in agreement with the lipogenic nature of the T3-regulated genes. Further analysis did not detect any transcriptional induction of SREBPs by T3. Instead, we showed that T3 decrease the concentration of ER-bound SREBP1, probably due to induction of its proteolytic processing. Regulation of SREBP activity by T3 seems to be complex and involves multiple mechanisms. The thyroid hormone receptor (TR) and active SREBP1c can cooperate to activate the transcription of a single gene even when their response elements are situated very far apart [29]. Moreover, direct interaction between TR alpha and the active form of SREBP1 has been demonstrated when their binding sites are closely located in the promoter of the chicken acetyl-CoA carboxylase-α gene [30]. Direct interactions between TR and ER-resident SREBPs are unlikely to be responsible for the effect observed in this study since the two proteins have different intracellular localizations [31]. Nevertheless, this possibility can not be completely discarded since 10% of TRs are found in the cytosol both in the absence or presence of T3 [31]. Non-genomic effects of T3 such as the activation of PI-3 kinase, MEK and STAT transcription factors have been reported in several systems [32]. Whether the rapid induction of SREBP1 processing by T3 is due to a non-genomic mechanism of T3 action, or not, remains to be demonstrated. SREBP proteolyticactivation in the Golgi is regulated by its interaction with SCAP and Insig-1 and -2 [33]. When sterols are present at high concentrations, the SCAP/SREBP complex is retained in the ER. When the sterol concentration is reduced, SCAP does not interact with Insig and the SCAP/SREBP complex exits the ER and is delivered to the Golgi where it is proteolytically cleaved. A role of SCAP as a monitor of the composition of the cytoplasmic leaflet of the ER membrane has been proposed [33]. Since T3 has been shown to bind the outer half of the lipid bilayer in reconstituted microsomes [34], there is a hypothetical possibility that T3 could bind ER membranes and regulate SCAP activity. It is important to notice that the concentration of the nuclear (short) form of SREBP1 was also reduced upon T3 treatment. Why this occurs simultaneously to the transcriptional induction of SREBP target genes is unknown but a recent publication indicates that transcriptionally active SREBP1 is rapidly targeted for proteosomal degradation [35]. If this mechanism is at play, one would expect the effects of T3 on lipogenic genes expression to be transient. This is indeed the case, most of the T3 effects on lipogenic genes can not be found after 5 days of hormonal treatment [10]. Future studies will clarify the importance of SREBPs for thyroid hormone liver actions.

## Conclusion

In summary, we have analyzed six different experiments concerning the hepatic actions of GH, T3, estrogen and gender. We could conclude that GH and estrogen are both important determinants of gender-related differences in hepatic gene expression, that GH and T3 have overlapping effects on the regulation of several lipogenic genes, and that some T3 effects in the liver may be mediated through the induction of proteolytic processing of SREBP1. Through EndoGED and its web-based interface, we have made available a large data set of transcript profiles related to the actions of several hormones in different *in vivo *and *in vitro *models. This resource is of special interest for the endocrine researcher offering the possibility of in depth exploration of hormonal transcriptional actions and interactions. In the same way, here exemplified by the analysis of hormone actions in liver, the actions of other hormones can be explored to generate testable hypotheses of relevance to endocrine research.

## Methods

### Experimental design

We have studied the effects of different hormones on liver gene expression patterns in various rat and mouse models. The experiments included in this study are listed in Table 1. They were conducted separately in different groups of animals, and by different persons in the laboratory. Some of the animal experiments have been described in previous publications: infusion of bovine growth hormone (bGH) in young male rats [8], infusion of human growth hormone (hGH) in old male rats [9], comparison of female and male rats [8], and the rapid effects of thyroid hormone (T3) treatment of hypothyroid mice [10]. Total hepatic RNA from individual animals was isolated using Trizol (Invitrogen, CA) and microarray hybridizations were performed on arrays larger than the ones described in the previously published studies. At least 4 statistically independent microarray measurements were used to characterize each physiological situation; with the exception of the experiment where thyroid hormone actions were studied where RNA pooled from 5 different mice was used. Other experiments included in this study have not previously been described. Primary hepatocytes were isolated from young male rats and cultured on matrigel as described previously[16]. The cells were grown in serum-free William's media E (Invitrogen, CA) supplemented with 55 μg/ml ascorbic acid (Sigma-Aldrich, MO), 100 IU/ml streptomycin and 1 μg/ml insulin (Sigma-Aldrich, MO) for two days before adding 100 ng/ml bGH (National Hormone and Peptide Program, A.F. Parlow, USA) to the media. GH-treated and untreated cells were harvested 24 hours later in Trizol (Invitrogen, CA), and RNA was purified according to the manufacturer's protocol. The procedure was repeated with cells isolated from different rats. In another experiment, 3 months old male rats were injected with 17-α-ethinylestradiol (5 mg/kg body weight; Sigma-Aldrich, MO) or vehicle 24 hours before sacrifice. Total RNA was isolated using Trizol (Invitrogen, CA). All animal experiments used in these studies were approved by the local ethical committee.

### cDNA microarrays, probe preparation and hybridization

The cDNA microarrays used in this study were produced in our lab, as described previously [7]. They have, however, been extended to comprise about 6200 clones, including clones from the TIGR Rat Gene Index, Research Genetics (Invitrogen, CA), and our own obtained through differential cloning experiments. The arrays were pre-hybridized in 1% BSA, 5XSSC and 0.1% SDS at 42°C for 1–2 hours, washed in milli-Q water, and dried immediately before the probe was applied. Total RNA was reverse-transcribed in the presence of Cy3- or Cy5-conjugated dUTP (PerkinElmer, MA) and purified as described previously [13]. In all studies except the one with T3-treated mice, RNA samples originating from livers of individual animals were labeled and each tester sample was hybridized against a control sample. In the T3-study, a pool of tester samples (from 6 different T3-treated animals) was hybridized against a pool of control samples due to limited availability of RNA. Dye-swaps were used in all studies to reduce systematic errors [36]. The final volume was adjusted to 25 μl with hybridization buffer consisting of 3.4XSSC, 0.3% SDS, 20 μg mouse Cot1 DNA (Invitrogen, CA), 20 μg polyA RNA, and 20 μg yeast tRNA. After heating at 98°C for 2 min and cooling to room temperature, the probe was added to the array and covered with a plastic cover slip (Grace Bio-Labs, OR). Hybridization took place at 65°C for 15–18 hours. The array was then washed and scanned with a GMS 418 scanner (Affymetrix, CA).

### Data processing and analysis

Data processing was performed essentially as described previously [13]. The software GenePix Pro (Axon Instruments, CA) was used to quantify the fluorescence intensity of each spot and the surrounding background. Automatic and manual flagging were used to localize absent or very weak spots (less than 2 times above background), which were excluded from analysis. The signal from each spot was calculated as the average intensity minus the average local background. We used a normalization method that takes into account and corrects for intensity-dependent artifacts in the measurements, the locally weighted linear regression (Lowess) method in the SMA package (Statistics for Microarray Analysis, available at ) [36]. SMA is an add-on library written in the statistical language R.

We next used EndoGED to extract all expressed hepatic genes from the included experiments concerning hormone treatment of rodents. In total, 6096 transcripts were expressed in one or more hybridizations. Hierarchical clustering using the TIGR Multiexperiment Viewer (MeV) software (available at [37]) was performed to explore and compare the different hormone treatments in the generated gene expression matrix. The euclidean distance was used as distance metric. We first compared the usage of ratios from all hybridizations with the usage of just the median log2 ratio for each set of replicated measurements (experiment group). These strategies gave similar results in the clustering, grouping the replicated measurements closely together. The median rather than the mean ratio was used to minimize the influence of outliers. Therefore, we calculated the median log2 ratio for each transcript within each experiment group, and used this ratio for further analysis. Genes that were detected in only half of the replicated measurements, or less, were excluded since we did not consider these measurements reliable. Included in the hierarchical clustering were only genes that had four or more median expression ratios from the six experiment groups (2518 genes). Using the same dataset with median ratios from each experiment group, we also calculated the correlation coefficient between the different experiment groups.

A statistical evaluation of differentially expressed genes was performed using the SAM (Significance Analysis for Microarrays) statistical technique [38]. This was done for each experiment group separately. A 5% false discovery rate (FDR) was used as cutoff. A gene expression matrix containing only genes affected by GH *in vivo *in a statistically significant manner was extracted from EndoGED. The T3-regulated genes in this list were identified by applying a log2 ratio cutoff of ± 1 (2-fold regulation) in each of the T3 hybridizations. We next used the web-based tool eGOn (developed at the Norwegian University of Science and Technology, available at ) to functionally classify the transcripts. With the two-sided one-sample binomial test implemented in eGOn, we compared the list of differentially expressed genes to all genes expressed in the T3 and GH experiments. The same software was also used to classify all T3-regulated genes (at least 2-fold up-regulation), and to compare them to all genes expressed in the experiments regarding T3. For the comparisons between gender differentiated genes and the effects of GH and estrogen, a mean ratio cutoff (log2 ratio treated/untreated >0.58, corresponding to at least 1.5-fold difference) was applied on top of the SAM statistical criteria.

### Brief description of EndoGED

The EndoGED system includes a Lab Information Management System (LIMS) to manage array fabrication, and modules to collect, store and process gene expression data concerning the actions of hormones. The system offers a flexible solution to integrate external analysis tools, including possibilities to store transformed expression values (e.g. normalized ratios) and associated parameters derived from statistical evaluation. The systems run on Microsoft operating system and have a client-server architecture implemented in SQL-Server as the database engine and Borland Delphi for the program modules. In the database design, we have taken into consideration the latest MIAME recommendations from the MGED Society regarding microarray data description [39]. We have implemented a detailed description of biomaterials and treatments used in the experiments, carefully considering what information would be significant to the endocrine researcher. The exploration tools have been implemented to allow easy retrieval of relevant data through a multilevel search engine. Furthermore, we have made part of our collected data available through Internet for easy access to researchers worldwide . The database structure and software are available for free to academic and other nonprofit researchers.

### Real Time-PCR

The expression of some genes from the array experiments were verified using quantitative real-time PCR. The Dynamo kit (Finnzymes Oy, Finland) containing SYBR Green was used for quantification. The primers and applied annealing temperatures are listed in Table 2. The expression of all genes was normalized to glyceraldehyde-3-P dehydrogenase (GAPDH), which was always measured in parallel to the other genes.

### Promoter analysis

We undertook a promoter analysis of a group of genes that were all up-regulated more than 2-fold two hours after T3 treatment in hypothyroid mice. We sought to find out if there was a statistically significant overrepresentation of SREBP and TRE binding sites in the T3-regulated group of genes compared to a control group, unaffected by the treatment. We used pair wise cross-species comparison (phylogenetic footprinting) as described by Lenhard et al [40] to identify putative transcription factor binding sites in the regions upstream of the transcriptional start site (TSS). Comprehensive reviews covering the field of transcriptional regulation bioinformatics are available [41,42].

As the study concerned mice, we used mouse orthologs to the rat genes printed on the arrays. To locate mouse orthologs for the rat sequences, we aligned the rat sequences to the mouse genome (NCBI build 30) using BLAT [43] and then searched for mouse cDNA sequences aligning to the same loci as the rat sequences and indicating similar gene structures. Human cDNA sequences orthologous to the mouse cDNA sequences were identified using the GeneLynx database [44,45]. Guided by genomic mappings of the cDNA sequences from the UCSC Genome Browser Database [46], we retrieved human (NCBI build 33) and mouse gene and promoter sequences. Since cDNA sequences are often truncated, we used consensus exon-intron structures derived from overlapping and similar cDNA mappings. We aimed to retrieve sequence from -5000 to +100 relative to TSSs. However, to ensure that the corresponding human and mouse genomic regions were extracted, we extended this region if, in an alignment of the human and mouse gene sequences, the TSSs could not be align. In addition, to avoid inclusion of other nearby genes in the retrieved sequences, we truncated the sequences at the border of any multi-exon cDNA mapping upstream of a TSS. If such a mapping was encountered within 100 bps of a TSS, no sequence was retrieved. Genes which could not confidently be mapped onto either mouse or human genomes were excluded from the analysis.

We analyzed 30 genes rapidly up-regulated by T3 (indicated with a * in Table 6) and, as a control group, 300 expressed but unaffected genes that were randomly selected from the array. We used a 50 bp sliding window and 70% sequence conservation for the alignment windows. The binding model was constructed by merging two matrix models (M00220 and M00221) from the TRANSFAC database [47]. The score cutoff for this model was set to 75%.

### Western blots

Whole cell protein extracts were prepared from frozen liver tissue by homogenization in RIPA buffer. Protein extracts were resolved by SDS-PAGE and transferred to PVDF membranes with a Trans-Blot SD semi-dry transfer cell (Hoefer, Pharmacia Biotech, Sweden). The filters were blocked for 1 h at RT in TTBS (20 mM Tris-HCl, 150 mM NaCl, Tween 0.1%, pH 7.4) containing 5% skim milk powder. Membranes were incubated overnight at 4°C with an anti-SREBP1 or antiβ-actin, as a loading control (SantaCruz Biotechnology, USA). After three 10 min washes in TTBS, binding of primary antibody was visualized using horseradish peroxidase-conjugated secondary antibodies, and the immunolabeling was detected by an enhanced chemoluminescence (ECL) method according to the manufacturer's instructions (Pierce Chemical Company, USA).

## Authors' contributions

NS, LH, P.T-E, LFP and A.F-M carried out the microarray studies, NS, A.F-M and RM implemented the EndoGED database. NS carried out the expression measurements by RT-PCR. AS, BL and PS carried out the promoter analysis. AF-M carried out the Western Blot analysis. All authors read and approved the final manuscript.

## Figures and Tables

**Table 1:** Experiments included in the study.

**species**	**age**	**sex**	**tissue**	**Control sample**	**Tester sample (hormone, time, dose)**
rat	3 months	M	liver	c.i. of vehicle	c.i. of bGH, 1 week, 5 μg/h
rat	2 years	M	liver	c.i. of vehicle	c.i. of hGH, 3 weeks, 0.34 μg/g body weight/day
rat	2 months	M	hepatocytes	medium	bGH added to medium, 24 hours, 100 ng/ml
rat	3 months	M/F	liver	male	female
rat	3 months	M	liver	vehicle	17-α-ethinylestradiol, 1 day, 5 mg/kg body weight
mouse	3 months	M	liver	hypothyroid	injection of T3 & TT4, 2 hours, 5 μg T3 + 5 μg T4

Expression profiles from six independent studies regarding gender differences and hormonal regulation of hepatic gene expression were included in the study. bGH = bovine growth hormone, hGH = human growth hormone, T3 = triiodothyronine, T4 = thyroxine, c.i. = continuous infusion, hepatocytes = primary rat hepatocytes. All rat experiments used Sprague Dawley rat strain. The mice strain used to study thyroid hormone actions was a hybrid of 129/Sv X C57Bl76J.

**Table 2:** The primers, amplicon sizes and annealing temperatures used for gene expression measurements by real-time PCR.

**gene**	**left primer**	**right primer**	**size**	**temp**
FAT/CD36	GCAACAACAAGGCCAGGTAT	TGTGGCTGAGCAGAAAGAGA	200	54
Ppp3ca	GCAGGCTGGAAGAAAGTGTC	AAGGCCCACAAATACAGCAC	200	54
Hsd11b1	TTTTGCAGAGCGATTTGTTG	TGCTCAGGACCACATAGCTG	200	54
Phyh	TACGTGGAGTGCTTCACTGG	CCATTGTTCCTGTCGATGTG	200	54
Srebp1a	GCGCCATGGACGAGCTG	TTGGCACCTGGGCTGCT	200	57
Srebp1c	GGAGCCATGGATTGCACATT	GCTTCCAGAGAGGAGGCCAG	200	54
Srebp2	CCCTTGACTTCCTTGCTGCA	GCGTGAGTGTGGGCGAATC	200	^54^

The expression of phytanoyl-CoA hydroxylase (Phyh), hydroxysteroid 11-beta dehydrogenase 1 (Hsd11b1), the catalytic subunit of protein phosphatase 3, alpha isoform (Ppp3ca), fatty acid translocase/cd36 antigen (FAT/CD36) and sterol regulatory element-binding proteins (SREBPs) 1a, 1c and 2 were measured using the primers shown in the Table. To allow comparison between samples, the expression data for each of the genes was normalized to the expression of glyceraldehyde-3-phosphate dehydrogenase (GAPDH), which was always run in parallel.

**Table 3:** Comparisons of GH response in liver and in primary hepatocytes

Genes	in vivo		hepatocytes	
	
	Array	RT-PCR	Array	RT-PCR
Phyh	0.34	0.6	0.54	0.6
Hsd11β1	0.31	0.28	0.63	0.5
Ppp3ca	1.33	1.43	1.48	1.33
CD36	2.39	2.78	1.26	2.07

Microarray and RT-PCR expression measurements for phytanoyl-CoA hydroxylase (Phyh), hydroxysteroid 11-beta dehydrogenase 1 (Hsd11β1), the catalytic subunit of protein phosphatase 3, alpha isoform (Ppp3cα) and fatty acid translocase/CD36 antigen (FAT/CD36). The real-time PCR results were normalized to GAPDH. The ratios between treated and untreated samples *in vivo *(young male rats treated with bGH or vehicle for 1 week via minipumps, n = 4 per group), and the ratios between GH-treated (24 hours) and untreated primary hepatocytes are shown.

**Table 4:** Genes with a gender-differentiated expression pattern that were also affected in male rats continuously infused with GH.

Unigene	Accession	Name	F/M	GH/untreated
Female predominant and up-regulated by continuous infusion of GH				
Rn.3211	AW917574	similar to TNF ligand superfamily member 12	4.61	8.14
Rn.14535	AI070587	carboxylesterase 2 (intestine, liver)	3.54	4.57
Rn.53990	AJ302031	alpha-1-B glycoprotein	7.08	3.60
Rn.2586	NM_031572	Cytochrom P450 15-beta gene	4.72	3.08
Rn.115975	AW916713	EST sequence	5.61	2.85
Rn.2011	AA818134	peroxiredoxin 3	8.75	2.78
Rn.107116	AW142960	EST sequence	4.66	2.77
Rn.101709	AA819200	similar to Alcohol sulfotransferase (Hydroxysteroid sulfotransferase)	9.73	2.71
Rn.92406	AA819605	rat senescence marker protein 2A gene, exons 1 and 2	9.17	2.67
Rn.11377	AA875291	HRAS like suppressor	2.13	2.61
Rn.3790	L19658	cd36 antigen	5.81	2.39
Rn.91378	AA818024	sulfotransferase, hydroxysteroid preferring 2	7.53	2.35
Rn.32282	AI045872	arginine vasopressin receptor 1A	1.86	1.99
Rn.1292	AA858662	tyrosine 3-monooxygenase/tryptophan 5-monooxygenase activation protein, zeta polypeptide	2.20	1.99
Rn.102325	AW917611	EST sequence	5.41	1.94
Rn.4000	X74402	guanosine diphosphate dissociation inhibitor 1	2.38	1.84
Rn.91122	AA858966	Cytochrome P450, subfamily IIC6	2.28	1.84
Rn.6946	AW140722	ferredoxin 1	2.28	1.79
Rn.8195	AW917572	EST sequence	3.61	1.68
Rn.106771	AA996745	similar to stromal interaction molecule 1	1.94	1.55
Rn.2180	CF108424	Atp5g3: ATP synthase, H+ transporting, mitochondrial F0 complex, subunit c (subunit 9) isoform 3	1.86	1.53
Male predominant and down-regulated by continuous infusion of GH				
Rn.1647	AF037072	carbonic anhydrase 3	0.06	0.09
Rn.37424	X79320	testosterone 6-beta-hydroxylase	0.10	0.15
Rn.23348	NM_031332	solute carrier family 22, member 8	0.10	0.16
Rn.103016	J00737	alpha-2u globulin PGCL1	0.01	0.18
Rn.106677	AA851893	similar to nucleoporin 37; nucleoporin Nup37	0.15	0.18
Rn.103770	CA504514	similar to Gelsolin precursor, plasma	0.19	0.22
Rn.888	AA819595	hydroxysteroid 11-beta dehydrogenase 1	0.12	0.31
Rn.7279	AF121345	phytanoyl-CoA hydroxylase (Refsum disease)	0.44	0.34
Rn.11320	CF110333	phosphoribosyl pyrophosphate synthetase 2	0.33	0.35
Rn.20403	CB805116	ectonucleotide pyrophosphatase/phosphodiesterase 2	0.22	0.36
Rn.106064	AW141056	similar to another partner for ARF 1	0.61	0.43
Rn.43232	AI045953	cysteine-sulfinate decarboxylase	0.27	0.54
Rn.22952	AW140875	putative homeodomain transcription factor 1	0.59	0.59
Rn.801	AW144321	EST sequence	0.48	0.59
Rn.6835	AA963739	similar to Putative lysophosphatidic acid acyltransferase	0.48	0.59
Rn.12345	AW916917	similar to RIKEN cDNA 6330575P11	0.48	0.64
Rn.93760	AA998734	glutathione S-transferase, mu 1	0.45	0.65
Male predominant but up-regulated by continuous infusion of GH				
Rn.29771	AA900486	ATP citrate lyase	0.46	2.61
Rn.10992	AA964628	glucose-6-phosphatase, catalytic	0.48	2.10
Rn.98269	NM_145878	fatty acid binding protein 5, epidermal	0.63	2.03
Rn.9486	X62888	fatty acid synthase	0.56	2.36

Differentially expressed genes were defined using SAM statistics, with a 5% false discovery rate as cutoff. An additional criterion was for the gene to have a mean ratio of at least 1.5. The table shows UniGene ID, GenBank accession number, gene name, and the median expression ratio (female/male or GH treated/untreated male).

**Table 5:** Genes with a gender differentiated expression pattern that were also affected in male rats treated with 17-α ethinyl-estradiol (EE).

UniGene	GBAccession	Gene Name	F/M	EE/untreated
Female predominant and up-regulated by ethinylestradiol				
Rn.40365	AA819200	hydroxysteroid sulfotransferase subunit	9.73	3.39
Rn.40124	AA819605	Rat hydroxysteroid sulfotransferase a (STa) mRNA, complete cds	9.17	3.13
Rn.2011	AA818134	Peroxiredoxin 3	8.75	4.34
Rn.2151	AA818024	Rat hydroxysteroid sulfotransferase mRNA, complete cds	7.53	3.93
Rn.53990	AJ302031	Rattus norvegicus mRNA for putative alpha 1B-glycoprotein (ORF1)	7.08	1.53
Rn.102325	AW917611	Similar to Kruppel-like factor 7 (ubiquitous)	5.41	1.50
Rn.2586	NM_031572	Rattus norvegicus Cytochrom P450 15-beta gene (Cyp2c12)	3.89	1.74
Rn.4000	X74402	R. norvegicus rab GDI alpha mRNA	2.38	1.77
Rn.1247	AA818043	cytochrome P450, 2c39	2.31	5.72
Rn.7245	AA858966	Rat cytochrome P450 PB1 (PB1 allele) mRNA	2.28	3.39
Rn.17105	AI029316	ESTs, Highly similar to tetrahydrofolylpolyglutamate synthase	1.88	3.74
Rn.32282	AI045872	R. norvegicus mRNA for V1a arginine vasopressin receptor	1.86	1.79
Rn.2382	AA964489	R. norvegicus mRNA for C-CAM2a isoform	1.79	3.47
Rn.13801	AW142659	Amphoterin induced gene and ORF 3	1.69	1.78
Rn.23741	AA900073	ESTs, Weakly similar to Ser/ Thr protein phosphatase 5	1.59	2.35
Female predominant and down-regulated by ethinylestradiol				
Rn.1292	AA858662	Tyrosine 3-monooxygenase/tryptophan 5-monooxygenase activation protein, zeta polypeptide	2.20	0.18
Rn.756	AA859785	Rat alcohol dehydrogenase (ADH) mRNA, complete cds	1.88	0.61
Rn.19721	U73174	glutathione reductase mRNA	1.64	0.43
	X12367	glutathione peroxidase I	1.69	0.56
Male predominant and down-regulated by ethinylestradiol				
Rn.1647	AF037072	carbonic anhydrase III	0.06	0.06
Rn.37424	U09742	CYP3A2 (testosterone 6-Beta-hydroxylase)	0.12	0.39
Rn.106677	AA851893	Similar to nucleoporin 37	0.15	0.16
Rn.102461	X16417	beta-globin	0.21	0.66
Rn.107334	NM_013096	Hemoglobin, alpha 1 (Hba1)	0.24	0.64
Rn.107335	AW142257	2-alpha globin; alpha-2-globin chain; hemoglobin alpha chain	0.28	0.59
Rn.100762	XM_235562	platelet-derived endothelial cell growth factor 1	0.42	0.33
Rn.7279	AF121345	peroxisomal phytanoyl-CoA hydroxylase (PHYH)	0.44	0.35
Rn.29771	AA900486	Rat ATP citrate-lyase mRNA, complete cds	0.46	0.32
Rn.15755	AW918421	EST	0.47	0.45
Rn.17644	AA817759	Peroxisomal Ca-dependent solute carrier-like protein	0.47	0.24
Rn.1086	AA817745	Adenylate kinase 4	0.52	0.57
Rn.5819	AA900928	Glutamic-oxaloacetic transaminase 1, soluble	0.53	0.58
Rn.9486	X62888	fatty acid synthase (EC 2.3.1.85)	0.56	0.34
Rn.48821	NM_012624	pyruvate kinase (L-type)	0.61	0.48
Rn.106064	AW141056	Similar to another partner for ARF 1	0.61	0.66
Rn.15739	M13508	apolipoprotein A – IV	0.62	0.53
Rn.10985	AW140851	choline kinase R; choline kinase R1	0.63	0.53
Rn.10389	AI058887	Rat p450Md mRNA for cytochrome P-450	0.65	0.54
Male predominant and up-regulated by ethinylestradiol				
Rn.66254	AA817793	Glucose-6-phosphatase catalytic subunit 3	0.68	1.55
Rn.888	AA819595	Hydroxysteroid dehydrogenase, 11 beta type 1	0.12	3.17

Differentially expressed genes were defined using SAM statistics, with a 5% false discovery rate as cutoff. An additional criterion was for the gene to have a mean ratio of at least 1.5. The table shows UniGene ID, GenBank accession number, gene name, and the median expression ratio (female/male or EE/Control male).

**Table 6:** T3 and GH have overlapping effects on gene expression in liver.

**Accession**	**Name**	**T3/untreated**	**young_GH**	**old_GH**		
AW140999	cytochrome P450 4A3	9.77	0.88	0.53		
AA956687	malic enzyme 1	7.86	2.21	2.64	*	#
BC029693	3-hydroxy-3-methylglutaryl-Coenzyme A synthase 1	6.82	1.42	1.79		
NM_145878	fatty acid binding protein 5, epidermal	6.59	2.02	1.46	*	#
K01934	thyroid hormone responsive protein	5.69	1.50	1.31		
AW142176	similar to Igh-6 protein	5.20	NA	1.70		
AW140621	similar to coenzyme A diphosphatase	4.94	0.90	1.37		#
AA925003	cytosolic acyl-CoA thioesterase 1	4.64	1.12	1.63	*	
AW916988	sterol-C5-desaturase (fungal ERG3, delta-5-desaturase)-like	4.51	1.43	1.50		#
AW142891	EST	4.41	1.10	1.18		
AA924590	FXYD domain-containing ion transport regulator 2	4.36	1.02	0.69		
BF281499	signal transducer and activator of transcription 5B	4.29	NA	1.07		
AA819712	EST	4.22	NA	1.13		
X16417	hemoglobin beta chain complex	4.13	0.91	1.25		
M34477	farensyl diphosphate synthase	3.96	2.01	1.50		#
NM_013096	hemoglobin, alpha 1	3.79	0.92	1.39	*	
AW916676	EST	3.79	0.91	1.53		
AW140468	hypothetical protein LK44	3.70	1.37	1.01	*	
AA923919	cathepsin E	3.63	NA	NA		
AW916686	similar to HT021	3.63	0.89	1.68	*	
AW142204	peptidyl arginine deiminase, type 4	3.59	0.92	1.44	*	
AA860063	similar to Glutathione S-transferase, theta 3	3.56	1.14	0.75		
X62888	fatty acid synthase	3.55	2.18	1.74	*	#
AW142682	podocalyxin-like	3.52	NA	NA		
AA957248	fatty acid elongase 1	3.50	1.08	1.20	*	
AA818305	similar to putative NAD(P)H steroid dehydrogenase	3.44	1.26	1.45		#
AA956747	fatty acid desaturase 2	3.33	1.11	1.51	*	
AW918434	hypothetical protein RMT-7	3.31	0.90	0.39	*	
AA997956	2,3-oxidosqualene: lanosterol cyclase	3.29	1.63	1.43	*	
CB736793	NADH dehydrogenase (ubiquinone) 1 alpha subcomplex 5	3.24	1.59	1.23		#
AW141045	multiple inositol polyphosphate histidine phosphatase 1	3.21	0.92	1.38	*	
AW142257	similar to hemoglobin alpha chain	3.19	0.93	1.45	*	
AW142786	Na+ dependent glucose transporter 1	3.17	0.98	1.22		
AA858817	EST	3.05	NA	0.94		
M31672	insulin-like growth factor binding protein 2	2.95	NA	1.08	*	
AA998020	protein O-mannosyltransferase 1	2.95	NA	NA		
AW913874	adipose differentiation-related protein	2.93	1.61	0.95	*	
AA924800	3-hydroxy-3-methylglutaryl-Coenzyme A synthase 1	2.88	NA	NA		
AA819200	similar to Alcohol sulfotransferase (Hydroxysteroid sulfotransferase) (ST) (ST-60)	2.86	3.01	2.44		#
AI045953	cysteine-sulfinate decarboxylase	2.83	0.54	0.61		
NM_012598	lipoprotein lipase	2.78	NA	NA	*	
AI029316	folylpolyglutamyl synthetase	2.72	1.31	1.07	*	
AA817840	opioid receptor, sigma 1	2.68	1.19	1.89	*	#
BF282623	EST	2.63	1.01	1.06		
AI137633	Nrp: neuropilin	2.59	NA	1.16		
AW914056	growth response protein (CL-6)	2.58	2.30	1.48		#
AI059131	2-4-dienoyl-Coenzyme A reductase 2, peroxisomal	2.56	NA	NA		
M73714	aldehyde dehydrogenase family 3, subfamily A2	2.55	0.96	1.20	*	
J02585	stearoyl-Coenzyme A desaturase 1	2.53	1.35	1.14		
NM_007760	carnitine acetyltransferase	2.51	NA	1.19	*	
AA819496	hypoxia induced gene 1	2.49	NA	0.79		
AW916795	occludin	2.49	0.94	1.01		
BF281801	kinesin family member 1B	2.44	0.68	1.28	*	
AF007775	aquaporin 8	2.42	1.21	1.86	*	#
AW916443	EST	2.32	0.95	0.92		
AW915619	inositol 1,4,5-triphosphate receptor 1	2.32	0.90	1.32	*	
AB012933	fatty acid Coenzyme A ligase, long chain 5	2.30	2.55	1.79		#
AA964037	phospholipase A2, group VI	2.26	NA	NA		
AI044427	similar to myotonic dystrophy protein kinase	2.17	NA	1.20	*	
AW913988	GTP cyclohydrolase 1	2.17	0.83	1.29	*	
M95591	farnesyl diphosphate farnesyl transferase 1	2.16	1.02	1.15	*	
AA998726	nasal embryonic LHRH factor	2.12	NA	0.92	*	
AW916626	EST	2.09	1.07	0.90		
AW140633	hemoglobin Y, beta-like embryonic chain	2.08	0.97	1.37		
AA926032	enoyl coenzyme A hydratase 1	2.05	0.91	1.19	*	
AA925091	fatty acid binding protein 4	2.01	NA	NA		
AA925731	isocitrate dehydrogenase 1	2.00	NA	NA	*	

All of the genes in this list were up-regulated by T3 in hypothyroid mice (ratio repeatedly larger than 2). Clones that did not map to any Unigene cluster were removed. The genes that were included in the promoter analysis, where mouse and human orthologs could be found, are indicated with a *. The genes that were up-regulated by continuous infusion of GH in at least one of the *in vivo *models in a statistically significant manner are indicated with a #. The table shows Unigene ID, GenBank accession number, gene name, and the median ratio (treated/untreated). "NA" means that the gene was flagged away in the analysis due to low expression in two or more hybridizations. The shown experiments are: T3: thyroid hormone treatment of hypothyroid mice for 2 hours; Young GH: infusion of bovine growth hormone (bGH) in young (3 months) male rats for 7 days; Old GH: infusion of human growth hormone (hGH) in old (2 years) male rats for 3 weeks.
